# Influence of Aging and Diabetes on the Mechanical Properties of Mouse Skin

**DOI:** 10.3390/dermatopathology12020018

**Published:** 2025-06-17

**Authors:** Sarah Miny, Gaël Runel, Julien Chlasta, Christelle Bonod

**Affiliations:** 1 Skin Functional Integrity Group, Laboratory for Tissue Biology and Therapeutics Engineering (LBTI), CNRS UMR5305, University of Lyon, 7 Passage du Vercors, 69367 Lyon, CEDEX 07, France; sarah.miny@ibcp.fr; 2 BioMeca, 69008 Lyon, France; gael.runel@bio-meca.com (G.R.); julien.chlasta@bio-meca.com (J.C.)

**Keywords:** skin, AFM, dermis, extracellular matrix, diabetes, glycation

## Abstract

Background: Diabetics accumulate Advanced Glycation End products (AGEs) such as Nε-(carboxymethyl)lysine (CML) in their skin, which can provoke changes in the skin’s biomechanical properties. The same changes are also observed during aging. Collagen is one of the first targets of glycation, and this leads to the disruption of the dermis, potentially contributing to the skin complications seen in diabetes, like impaired wound healing and the formation of chronic ulcers. We therefore investigated whether it was possible to detect differences in the biomechanical properties of the reticular dermis by comparing C57/BL6 control mice, type 1 and type 2 diabetic mice, and aged mice. Methods: To investigate this, we used an Atomic Force Microscope (a type of local probe microscope used to visualize the surface topography of a sample) to measure the elastic modulus of each skin sample. The elastic modulus is a parameter that describes a tissue’s resistance to elastic deformation when stress is applied. We also determined whether diabetes is associated with the accumulation of AGEs via Western blots. Results: We found that type 2 diabetic mice and aged mice had a stiffer reticular dermis than young control mice. No differences were found in type 1 diabetic mice. The results of the Western blot did not reveal any significant differences in the CML content in different types of mice, although a non-significant increase was found in type 2 diabetic and aged mice. We show that there is a significant positive correlation between the amount of CML in a mouse and the rigidity of its reticular dermis. Conclusions/interpretation: We have demonstrated that increased glycation in mouse skin is correlated with the biomechanical properties of that skin, which explains the wound healing defects diabetic patient’s experience. AFM is therefore a powerful technique that could be used to characterize the mechanical effects of treatments aimed at reducing the level of AGEs in the skin.

## 1. Introduction

Aging impacts the function of all organs, tissues, and cells, causing irreversible changes in their mechanical behavior. The skin, the largest organ in the body and its barrier against external aggression, is not exempt from this aging process [1]. There are two types of skin aging. Intrinsic aging, also called chronological aging, is an inevitable natural process caused by time, hormones, and genetic factors. It is characterized by modifications of the skin’s mechanical properties, which are supported by defects in skin renewal. Extrinsic skin aging, on the other hand, is caused by lifestyle and environmental factors such as UV exposure or pollution, as well as poor sleep and diseases such as type II diabetes [2,3].

The International Diabetes Federation estimates that 537 million adults (aged 20–79) worldwide had diabetes in 2021, and this number could rise to 783 million by 2045, making diabetes a major public health problem. It is a chronic disease characterized by an excess of sugar in the blood, known as hyperglycemia. Chronic hyperglycemia leads to microvascular, neuropathic, and skin complications. Diabetics suffer from a delay in wound healing, and one in ten will end up having a toe, foot, or leg amputated [4].

In diabetes, glycation, a non-enzymatic reaction between sugars and proteins known as the Maillard reaction [5], leads to the significant accumulation of Advanced Glycation End products (AGEs) in tissues such as the skin [6,7]. Members of the α-oxoaldehyde family, which include glyoxal, methylglyoxal, and 3-deoxyglucosone (3-DG), are known to be agents that lead to glycation reactions [8]. Glyoxal is found in large quantities in the plasma of diabetic patients, and it is responsible for the formation of a particular AGE called Nε-(carboxymethyl)lysine (CML) [3,5,6,7]. Interestingly, Mera et al. reported that the formation of AGEs in vivo may contribute to diabetic complications [9]. Collagen, which is a protein with a slow turnover rate, is particularly prone to glycation and can be cross-linked by glyoxal [10]. Additionally, it has been shown that other proteins and also nucleotides can be damaged by glyoxal [6] and that there is a strong accumulation of CML in the skin of diabetic patients, as measured in 94 volunteers using fluorescence and Raman spectroscopy [11]. In skin, glycation is characterized by the accelerated chemical aging of long-living tissue proteins [12] such as elastin [13]. It was also shown by Atomic Force Microscopy (AFM) that the surface of collagen fibrils was rougher and fiber bundles were stiffer and harder in aged dermis compared to young dermis [14]. Finally, the accumulation of AGEs could affect the behavior of collagen fibrils [15].

During skin’s aging, modifications occur in the two main superficial layers. The thickness of the epidermis decreases due to the reduced capacity for keratinocyte’s proliferation and differentiation, resulting in a change in their environment. The cellular composition and matrix organization of the dermis are also altered [16,17]. The number of fibroblasts decreases, leading to a reduction in the number of collagen and elastin fibers in the extracellular matrix [16,17]. It was also recently shown that glycation induces a decrease in the proliferation and migration of fibroblasts and that collagen is synthetized but not deposited in the extracellular matrix [18]. Each part of the skin has its own mechanical environment, which can be described by its rigidity. This specific mechanical environment is essential for proper tissue function. Atomic Force Microscopy (AFM) is a powerful technique for studying the mechanical properties of cells, tissues, and organs [19,20,21], and it can be applied to the characterization of the mechanical properties of skin subject to physiological or pathological conditions. The principle of AFM is based on the displacement of a probe, i.e., a tip, on the surface of a sample. AFM uses piezoelectric crystals to precisely record the displacement of the tip in three dimensions (x, y, and z), offering an extremely fine resolution [22]. The probe scans the sample’s surface, enabling the acquisition of high-resolution three-dimensional topographical images from which quantitative measurements, such as surface roughness and height profiles, can be derived. Beyond imaging, AFM also enables the measurement of the elastic modulus (expressed in pascals, Pa) of biological samples. This parameter characterizes a tissue’s resistance to elastic deformation under applied stress and is often used as a measure of a sample’s stiffness. To obtain this measurement, a controlled force is applied to the probe, causing it to indent the sample’s surface. The indentation depth varies according to the mechanical properties of the tissue, and the resulting depth variations are converted into force–indentation curves, from which the elastic modulus is extracted for each measured point within the scanned area.

In skin biology, AFM can be used on different types of samples: skin explants, longitudinal or transverse sections of skin, and primary cells isolated from skin explants. AFM measurements of sections of human skin from the dermis have revealed that they have different stiffness profiles depending on their depth in the dermis [23]. It has also been shown that skins exposed to UV, a factor involved in extrinsic skin aging, see their elastic modulus at a dermal level decrease compared to control skins [23]. This difference in stiffness at different dermal depths has also been observed in longitudinal sections of skin from male control mice [24].

In this study, we compared, by AFM, the biomechanical properties of the reticular dermis of skin from wild-type type 1 diabetic mice, a model of type 2 diabetes (db/db mice), and aged mice. We also explored the correlation between skin stiffness’s modulation and glycation of the extracellular matrix, with the aim of proposing a model of skin aging based on the biomechanical properties of glycated skin.

## 2. Materials and Methods

### 2.1. Animals

This study was conducted on 12-week-old C57BL/6 mice (*n* = 4), 19-month-old C57BL/6 mice (*n* = 3), 12-week-old type 1 diabetic C57BL/6 mice (*n* = 4), and 12-week-old type 2 diabetic C57BL/6 mice (db/db mice) (*n* = 5) (Janvier labs, Saint-Berthevin, France). All animals used in this study were male mice. The animals were kept at a controlled temperature (22 ± 2 °C) and humidity (60 ± 5%), with a 12 h light/dark cycle and ad libitum access to water and food. All procedures were carried out in accordance with the guidelines for the ethical care of experimental animals of the European Community and approved by scientific and ethics committees (agreement C2EA015, n°26546, Rhône-Alpes ethics).

### 2.2. Induction of Diabetes

After an 8 h fast, type 1 diabetes was chemically induced in a subgroup of the mice via a single intraperitoneal injection of streptozotocin (STZ) (Zanosar^®^, Keocyt, Montrouge, France; at a dose of 180 mg/kg) (protocol adapted from Furman, 2021, city, and country) [25]. Mice were considered diabetic when their blood glucose level was ≥300 mg/dL three days after the injection. Two units of insulin (Caninsulin, Intervet, Beaucouze, France) was administered to the diabetic animals every two days, and their body weight was monitored each week. Experiments were conducted four weeks after the induction of diabetes.

db/db mice are used as a model for type 2 diabetes and obesity. These mice have a homozygous mutation in the gene coding for the leptin receptor (Lepr^db^), which means that they never reach satiety. The day of the experiment, their blood glucose level was >400 mg/dL, which confirms their diabetic phenotype.

### 2.3. HES Staining

Skin samples were fixed in Alcool-Formol-Acetic acid liquid (Carbo Erba Reactifs, Val-de-Reuil, France) and embedded in paraffin. The staining of 5 µm sections of skin was conducted using hematoxylin, eosin, and safranin. Observations were made using an ECLIPE Ti-E inverted microscope (Nikon, Boston, MA, USA) equipped with a DS-U3 color camera (Amstelveen, The Netherlands) and NIS element imaging software (Nikon).

### 2.4. Atomic Force Microscopy Measurements

Skin samples were embedded in optimal cutting temperature (OCT) compound and cooled in liquid nitrogen. A Leica cryostat CM3050S (Leica, Wetzlar, Germany) at −20 °C was used to create 16 µm sections, which were placed on SuperFrost Plus slides (#22-037-246, Thermo Fisher, Waltham, MA, USA) and stored at −20 °C.

AFM indentation experiments were carried out using a Resolve Bioscope (Bruker Nano Surface, Santa Barbara, CA, USA) mounted on an inverted optical microscope (DMI8, Leica, Wetzlar, Germany) equipped with a 40× objective. A Nanoscope V controller with Nanoscope software version 8.15 was used. All quantitative measurements were performed using a standard conical tip (DNP-10A, Bruker AFM probes, Inc., Camarillo, CA, USA). The tip radius given by the manufacturer was 40–60 nm. The spring constant of the cantilever was measured using the thermal tune method and was 0.35 N/m [26].

Each AFM experiment consisted of acquiring a matrix of force–indentation curves at the level of the reticularis dermis. Force curves were acquired using the AFM QNM (Quantitative Nanomechanical Mapping) mode. The force measurements were performed in 1X PBS (#10010023, Gibco, Paisley, UK) on cryosections previously fixed with 4% formaldehyde (#J60401.AK, Thermo Fisher, Waltham, MA, USA) and labeled for DAPI (#D9542, Sigma, Saint Louis, MO, USA). Each AFM measurement consisted of the acquisition of 1024 force curves extracted over a 25 × 25 µm square with an indentation up to 5 µm. The measurement was acquired at 10 hz, with a ramp size of 8 µm and a trig threshold of 50 nm.

For the stiffness measurement, the Sneddon mathematical model was used to extract quantitative data on the elastic modulus (Ea) in pascals (Pa) from each curve, covering an indentation range of 0 to 5 µm on BioMeca Analysis processing software (version number 1.3.7).

The Sneddon model assumes a rigid cone is indenting a flat surface with the force (*F*) from the force curve, which is given by the formula

$$F=\frac{2}{\dot{\pi }}\cdot \frac{E}{1-v^{2}}\cdot tan\left(\alpha \right)\cdot \delta^{2}$$

*F* is the force from the force curve, *E* is Young’s modulus, ν is Poisson’s ratio, *α* is the half-angle of the indenter, and *δ* is the indentation.

Pascal (Pa), the derived unit of the International System of Units (SI) used to measure pressure or mechanical stress, is defined as one newton (N) of force applied uniformly over an area of one square meter (N/m^2^). In materials mechanics, the elastic modulus (or Young’s modulus), expressed in Pa, quantifies a material’s resistance to elastic deformation under applied stress and corresponds to the slope of the linear region of its force–indentation curve. Often referred to as “stiffness,” this parameter reflects a material’s ability to resist reversible deformation when subjected to a force. During an AFM measurement, a controlled force is applied to indent the probe into the sample’s surface, and the indentation depth (height) is recorded. The contact area depends on this indentation depth, and dividing the applied force by the contact area yields the mechanical stress. The elastic modulus is then calculated by dividing this stress by the resulting strain, thus making its unit the Pa.

### 2.5. Protein Extraction and Immunoblotting

Dorsal skin samples (5 mg) from mice were placed in a tube on ice and homogenized in Laemmli buffer (62.5 mM Tris-HCl, pH 6.8, 0.01% bromophenol blue, 10% glycerol, 2% SDS, and 50 mM DTT) supplemented with a protease and phosphatase inhibitor cocktail (#A32961, Thermo Fisher, Waltham, MA, USA). Two stainless steel beads were added to each tube, and the lyse was performed using a TissueLyser II system (#9003240, QIAGEN, Hilden, Germany) under the following conditions: two cycles of 2 min at 20 Hz followed by one cycle of 3 min at 30 Hz. Samples were subsequently kept in a chilled adapter at −20 °C.

The tubes were then centrifuged at 1500 rpm and 4 °C for 10 min, and the supernatants were carefully collected and kept on ice for further analysis. It is important to note that the use of Laemmli buffer prevents the reliable quantification of protein. Indeed, interactions between the Laemmli buffer components and the BCA Protein Assay Kit reagents (#23225, Pierce Technologies, Waltham, MA, USA) lead to the solution having a dark coloration, making absorbance measurements and the subsequent determination of protein concentrations impossible.

The proteins were denatured for 5 min at 95 °C in SB-DTT 5X, and 10 µL of the proteins was separated from the solution, according to protein size, by SDS polyacrylamide gel electrophoresis (10% resolving gel and 4% stacking gel) using stain-free reagent (#T54801, Sigma, Saint Louis, MO, USA) and 1X Tris-Glycine Sodium Dodecyl Sulfate buffer (TG-SDS). Before transfer, the gel was exposed to UV light to activate its stain-free signal using the Fusion Fx system (Vilber Lourmat, Collégien, France). The proteins were then electroblotted onto polyvinylidene difluoride (PVDF) membranes (#IPVH00010, Merck Millipore, Ireland) in TG-20% ethanol for 2 h at 220 mA and 4 °C. The membranes were then incubated in ECL reagent for 5 min (#34580, Thermo Fisher Scientific, USA), and the total protein, visualized using the stain-free system, was detected by chemiluminescence. The signal was recorded using a Fusion Fx system. The PVDF membranes were then blocked for 1 h using 1X Tris-buffered saline containing 0.1% Tween 20 and 5% non-fat milk (TBS-T milk). The primary antibody, rabbit polyclonal anti-carboxymethyl-lysine (#ab27684, Abcam, Cambridge, UK), was diluted to 1:500 in 1X TBS-T milk and incubated overnight at 4 °C. After washing them, the membranes were incubated for 1 h at room temperature using goat anti-rabbit HRP conjugate (#ab6721, Abcam, Cambridge, UK) diluted to 1:10,000 in 1X TBS-T milk. The membranes were then incubated in ECL reagent for 5 min, and the antigens were detected by chemiluminescence. The signal was recorded using a Fusion Fx system (Vilber Lourmat, Collégien, France). The total CML signal for each lane was quantified and normalized to the corresponding total protein signal visualized by the stain-free system.

### 2.6. Statistical Analysis

Data are expressed as mean ± SD. Based on the normality of the data (Shapiro–Wilk test), statistical significance was calculated by a one-way analysis of variance (ANOVA) and a Pearson correlation using Prism software (version 10, GraphPad Software, San Diego, CA, USA). When the data lacked normality, a Mann–Whitney test was used. Mean differences were considered statistically significant when *p* < 0.05: *—*p* < 0.05; **—*p* < 0.01.

## 3. Results

### 3.1. Mechanical Properties of Skin

**Figure 1:**
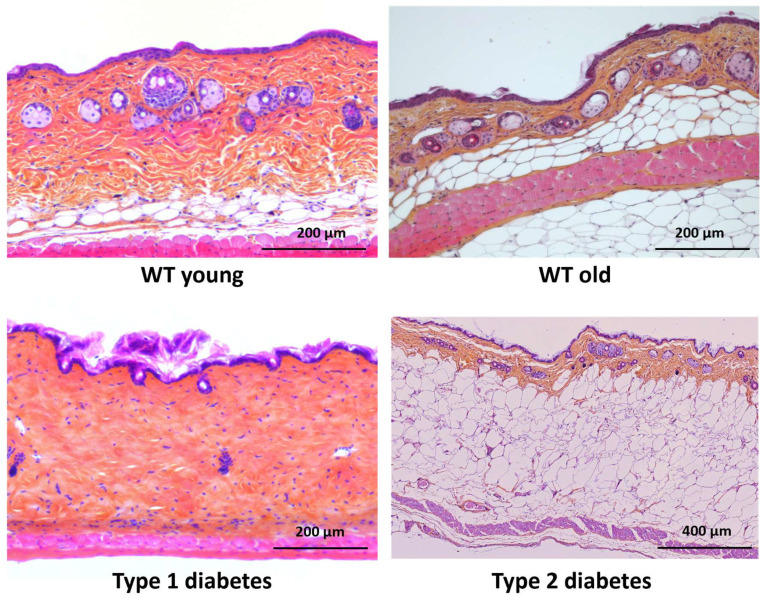
Histological analysis of mouse skin via HES staining. Skin samples from young, old, and diabetic mice were fixed and embedded in paraffin. 5 µm sections of skin were stained using hematoxylin, eosin, and safranin allowing the visualization of epidermis, dermis and hypodermis. s. As expected, these images show a total absence of adipose tissue in type 1 diabetic mice and, on the contrary, a strong accumulation of hypodermis in type 2 diabetic mice. Scale bar = 200 mm for young, old, and type 1 diabetes; scale bar = 400 mm for type 2 diabetes.

In order to characterize the differences between mouse skin samples, we first analyzed the morphology of the skin in young control mice, old control mice, and type 1 and type 2 diabetic mice at the histological level. As expected, we observed a decrease in adipose tissue in type 1 diabetic mice and a strong accumulation of adipocytes in type 2 diabetic mice (Figure 1).

**Figure 2:**
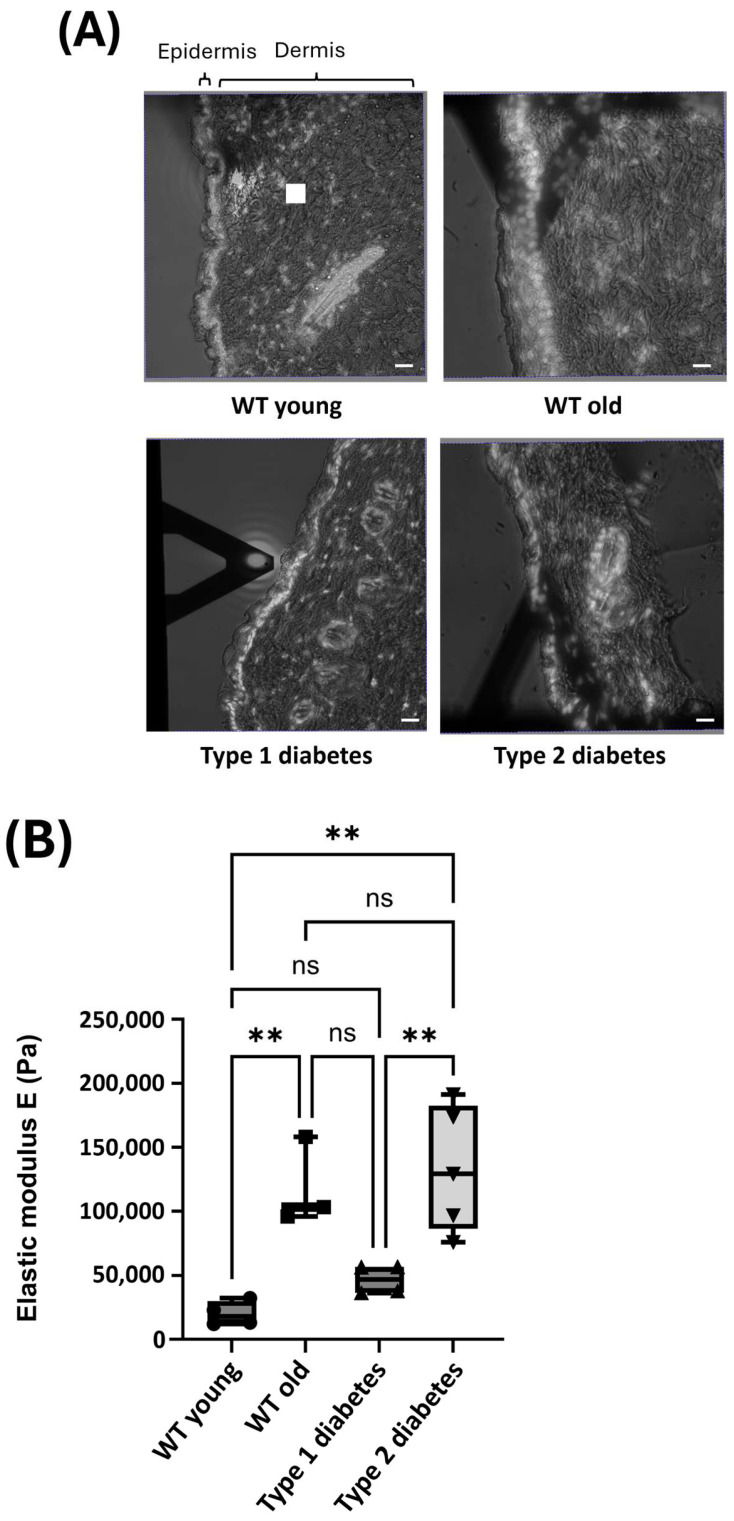
Analysis of average reticular stiffness measured by AFM on frozen skin sections from WT young (*n* = 4), WT old (*n* = 3), type 1 diabetic (*n* = 4), and type 2 diabetic mice (*n* = 5) mice. For the AFM measurements, three independent areas were assessed for each mouse, with each 25 × 25 µm area measured 10 times at the level of the reticular dermis. (**A**) Representative tissue sections from each type of mouse. The sections were stained with DAPI (white dots) for fluorescence labeling, which was used to aid the orientation of the tissue and clearly distinguish the epidermis from the dermis. The images presented are merged DAPI fluorescence and brightfield images of the tissue sections. As such, the epidermis and dermis are identified using the brackets above the image. An example of the AFM measurement area is represented by the white square in the section of representative tissue from a young WT mouse. (**B**) Box plot illustrating the average reticular dermis stiffness of all mice. A one-way ANOVA with Tukey’s multiple comparison test was employed to compare the reticular dermis stiffnesses of all mice. ns = not significant; ** *p*-value < 0.01.

We then used AFM to determine the stiffness of the reticular dermis. First of all, sections were stained with DAPI (Figure 2A, white dots), which was used exclusively to help orient the tissue and clearly distinguish the epidermis from the dermis (as highlighted by the brackets in Figure 2A). AFM measurements were then performed at the dermis level, from the location indicated by the white square on the section of skin taken from a WT mouse (Figure 2A).

The stiffness of the reticular dermis was considered in relation to several factors, including fibroblast density and the composition and organization of the ECM. The elastic modulus, expressed in Pa, enabled us to compare the dermal rigidity of different skin types. The elastic modulus of the skin from young control mice is around 20,000 Pa. In comparison, the elastic modulus observed in old mice and type 2 diabetic mice is significantly higher, 119,000 and 133,000 Pa, respectively. In type 1 diabetic mice, the elastic modulus tends to be higher than that of young control mice but not significantly different. Finally, no significant difference was observed between the elastic modulus of old and type 2 diabetic mice (Figure 2B).

We can see that old mice had a higher reticular dermis stiffness than young mice, meaning that there is an increase in the stiffness of the skin during aging. As the type 2 diabetic mice also displayed an increased stiffness in their reticular dermis compared to the young control mice, type 2 diabetes can be said to increase the stiffness of the reticular dermis to a similar extent as aging. Finally, type 1 diabetes also seems to increase the stiffness of the reticular dermis.

### 3.2. Glycation and the Mechanical Properties of Skin

As glycation is a factor involved in the changes in skin mechanics seen during aging and in type 1 and type 2 diabetes, we sought to quantify the amount of CML in the skin of young and old control mice, as well as type 1 and type 2 diabetic mice. For this purpose, Western blots of CML were performed.

**Figure 3:**
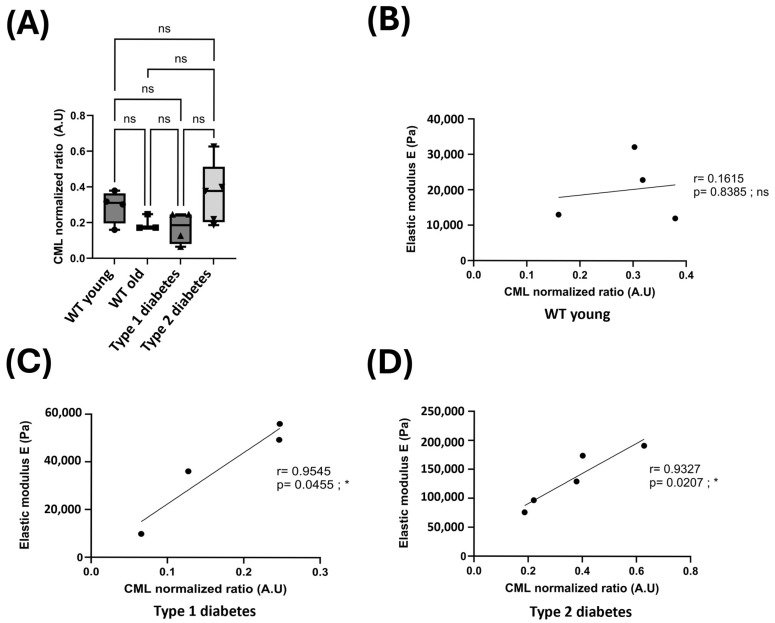
(**A**) Quantification of the CML levels normalized to the total protein levels extracted from the skin of WT young (*n* = 4), WT old (*n* = 3), type 1 diabetic (*n* = 4), and type 2 diabetic (*n* = 5) mice. A one-way ANOVA with Tukey’s multiple comparison test was employed to compare normalized CML levels between all mice. ns = not significant; * = significant. (**B**–**D**) Reticular dermis stiffness of WT young (**B**), type 1 diabetic (**C**), and type 2 diabetic (**D**) mice according to their normalized CML ratio. A Pearson correlation was performed to evaluate the correlation of reticular dermis stiffness with CML level.

The results of the Western blot did not reveal any significant differences between the various types of mice (Figure 3A and Figure S1). Indeed, the mean relative quantification of CML appears similar between young, old, type 1, and type 2 diabetic mice. Although the difference was not significant, an increase in the mean amount of CML in the skin of type 2 diabetic mice compared to young control mice was observed. Likewise, although the difference was not significant, aged and type 1 diabetic mice exhibited a lower mean amount of CML in their skin than young control mice.

In order to study the correlation between the presence of CML and the rigidity of the reticular dermis, Pearson correlation tests were performed. In young control mice, the Pearson test did not reveal any correlation between the two factors (Figure 3B). On the other hand, in type 1 and type 2 diabetic mice, it revealed a significant positive correlation between the amount of CML and the rigidity of the reticular dermis. Indeed, the higher the amount of CML, the greater the rigidity of the reticular dermis (Figure 3C,D).

To conclude, although we did not observe significant differences in the amount of CML present in different mice, we observed a positive correlation between the amount of CML and reticular dermis stiffness in type 1 and type 2 diabetic mice.

## 4. Discussion

The objective of our study was to investigate the mechanical properties of mouse skin during intrinsic aging and in a pathological context. To achieve this, we studied the rigidity of the dermis in young and old control mice and type 1 and 2 diabetic mice using Atomic Force Microscopy. During aging and in diabetes, the skin accumulates AGEs that can impact the organization of its extracellular matrix. So, our second objective was to study whether there was a correlation between the presence of glycation products and the rigidity of the dermis under different conditions.

The analysis of the elastic modulus of mouse skin sections using AFM allowed us to demonstrate that the rigidity of the reticular dermis increases during aging. This observation aligns with the previously documented mechanical modifications of the skin during aging [14,27,28].

In this study, we also demonstrated that there is an increase in the rigidity of the reticular dermis in the pathological context of type 1 and type 2 diabetes. This observation is consistent with previous studies that have highlighted a decrease in the elasticity of glycated skin, as well as modifications of the organization and composition of the ECM in diabetic skin [29,30].

In contrast, the increase in skin rigidity in type 1 diabetic mice is significantly less than that observed in control mice. This result may be explained by the fact that type 1 diabetic mice begin to develop diabetes from the age of eight weeks via STZ injection, which is probably not old enough for the diabetes to have developed sufficiently [31].

To assess the correlation between skin glycation and skin stiffness, we studied the amount of CML present in the skin by conducting Western blots for each of the mice whose skin stiffness was assessed.

Interestingly, our results indicate that the average amount of CML present in the skin of mice does not increase during intrinsic aging. These results are in contrast to studies reporting an increase in AGEs, including CML, during aging in human and murine skin [7,32,33]. This divergence can be explained by individual variations in the mechanisms regulating AGEs, and particularly the differences in the metabolic rates of glycation and deglycation among skin donors. Given that the older mice used in this study were 19 months old, it is possible that their aging phenotypes were not fully developed. Additionally, an abnormal thickness of the epidermis was observed in the older mice, which leads to further uncertainty regarding the aging phenotype of these mice.

Regarding type 2 diabetic mice, their skin contained a higher quantity of CML compared to the skin of control mice, confirming an increase in glycation in type 2 diabetic skin [34]. However, type 1 diabetic skin did not contain a significantly different quantity of CML compared to that in control mice. Although it is accepted that type 1 diabetic mice have higher sugar levels in their blood, it is possible that the type 1 diabetic phenotype was not fully established in their skin, particularly in terms of the accumulation of AGEs. This raises questions about the timeline for the establishment of the type 1 diabetic phenotype in mouse skin.

Ultimately, our results show that there is a positive correlation between the amount of CML in the skin and its rigidity. This correlation is particularly significant in type 2 diabetic mice, which have a high level of AGEs, including CML. Indeed, the more CML present in type 2 diabetic mice, the greater the rigidity of their skin. This phenomenon can be explained by the cross-linking of collagen fibers induced by AGEs. These bonds interfere with the organization of the collagen in the ECM and the contractile capacity of fibroblasts, which can thus lead to changes in the mechanical properties of the skin [29,35].

This study has highlighted a stiffening of skin affected by type 1 and 2 diabetes, which is similar to the stiffness observed in aged skin. Moreover, it has been demonstrated that there is a positive correlation between the level of AGEs in the skin and its stiffness. Given that the biomechanics of the skin greatly influence the proliferation, differentiation, and metabolism of the cells composing the skin, the demonstration of this correlation in type 1 and 2 diabetic mice may provide insights into the structural, metabolic, and healing defects of diabetic skin.

Atomic Force Microscopy (AFM) is a powerful and increasingly popular technique for studying the mechanical and topographical properties of biological tissues, particularly skin. It can probe nanoscopic features such as stiffness, elasticity, surface texture, and cell–cell interactions. Using treatments aimed at reducing the level of AGEs in human diabetic patients may be a viable solution that could reduce the mechanical changes observed in the skin of diabetic mice, and AFM is an effective tool for characterizing the mechanical effects of those treatments. Finally, AFM makes it possible to study the biomechanics of the skin in a large number of pathologies such as atopic dermatitis [36], epidermolyis bullosa [37], pemphigus vulgaris [38], alopecia [39], and Ehlers–Danlos syndrome [40].

## 5. Conclusions

We are the first to demonstrate a correlation between the biomechanical properties of diabetic mouse skin and increased glycation, which may help explain the impaired wound healing observed in diabetic patients. AFM has thus been proven to be a powerful technique for characterizing the mechanical impact of treatments aimed at reducing the accumulation of Advanced Glycation End products (AGEs) in the skin.

Our findings have potential applications not only in the study of the aging of skin and complications related to diabetes but also in the evaluation of new therapeutic molecules designed to enhance skin healing.

## Data Availability

Derived data supporting the findings of this study are available from the corresponding author [CB] on request.

## Supplementary Materials

The following supporting information can be downloaded at: https://www.mdpi.com/article/10.3390/dermatopathology12020018/s1, Figure S1: Quantification of the CML amount by Western-blot (upper images) and analysis of the total protein amount from skin by stain-free system (lower images).

## Abbreviations

The following abbreviations are used in this manuscript:

ECM	Extracellular Matrix
AGEs	Advanced Glycation End Products
AFM	Atomic Force Microscopy
