# Supplementary material for: Influence of Aging and Diabetes on the Mechanical Properties of Mouse Skin
Source: Dermatopathology (Basel). 2025 Jun 17;12(2):18. doi: 10.3390/dermatopathology12020018 (PMC12192205; doi:10.3390/dermatopathology12020018)

T2D : Type 2 diabetes
T1D : Type 1 diabetes
Old : WT old
Young : WT young

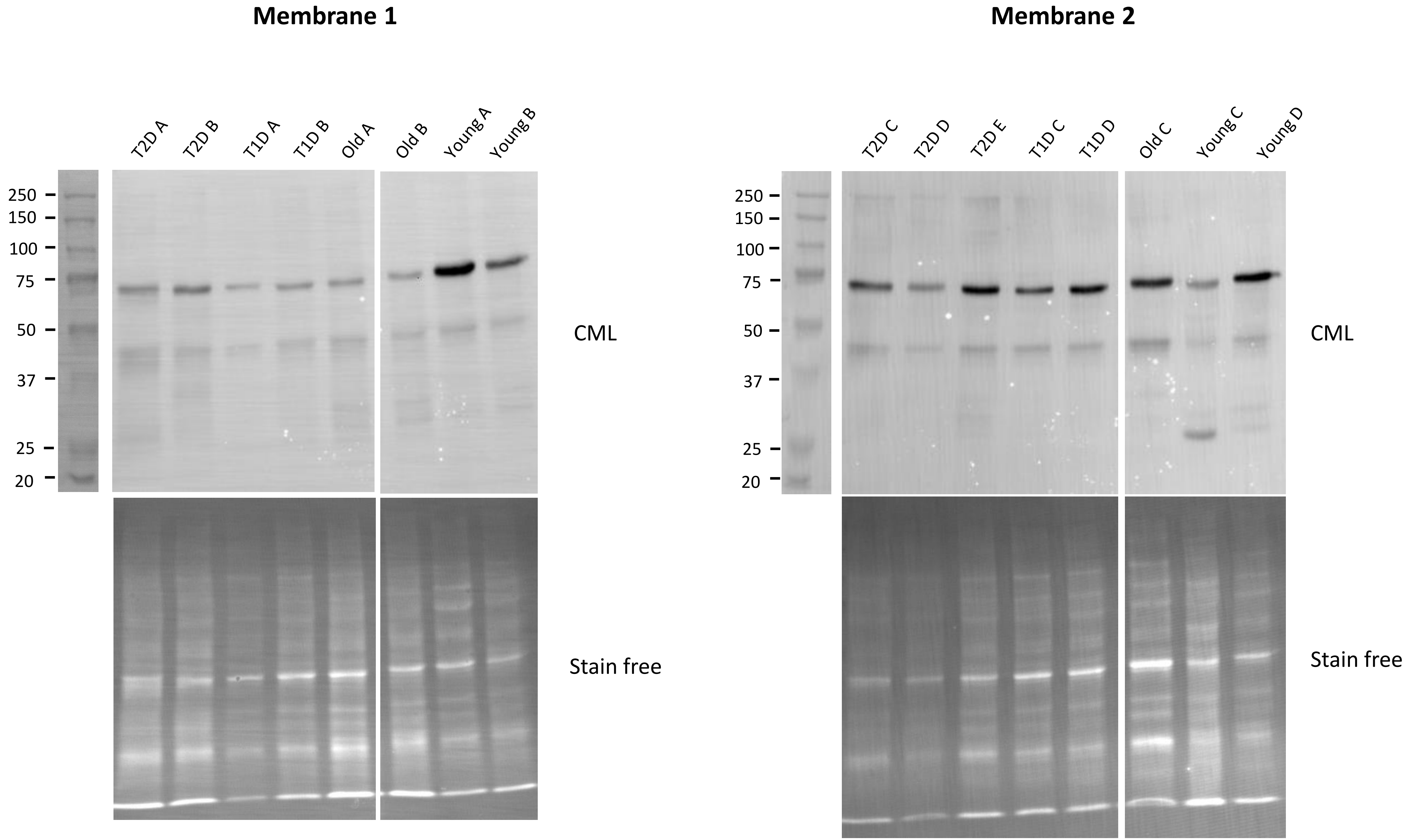

Membrane 1

The layout of a Membrane 1

T2D A	T2D B	T1D A	T1D B	Old A	//	Old B	Young A	Young B

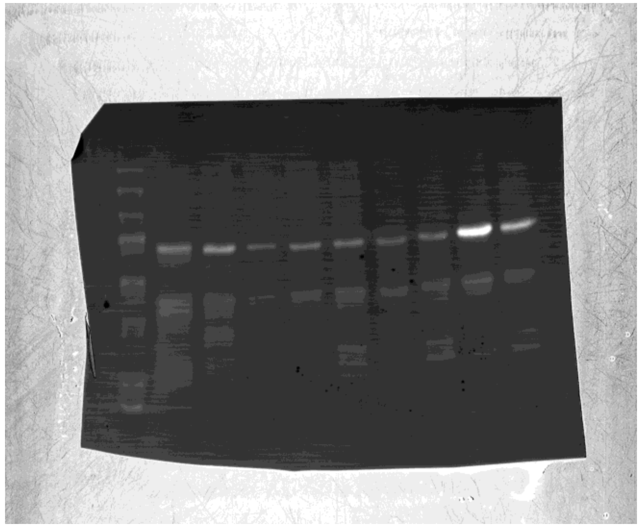

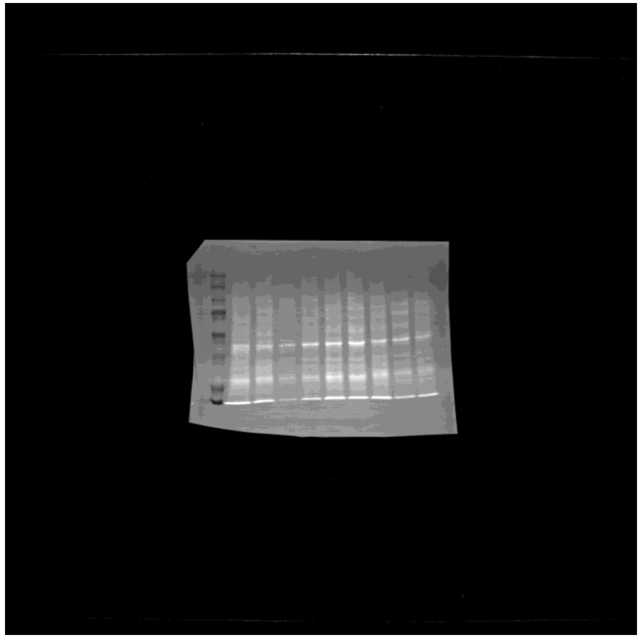

Originals pictures

Membrane 2

The layout of a Membrane 2

T2D C	T2D D	T2D E	T1D C	T1D D	//	Old C	Young C	Young D

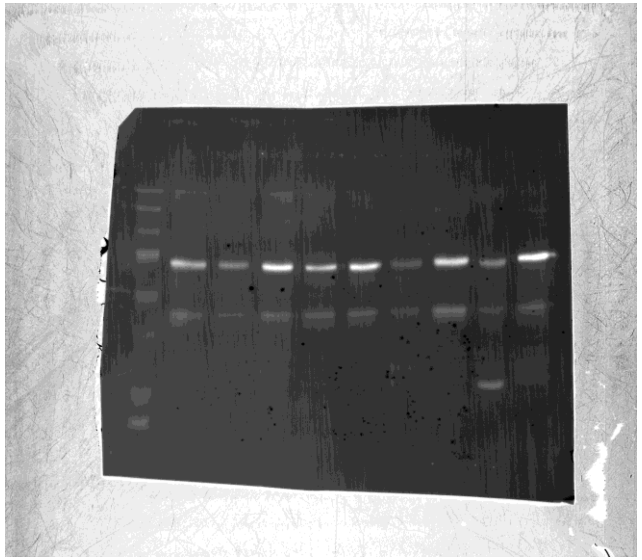

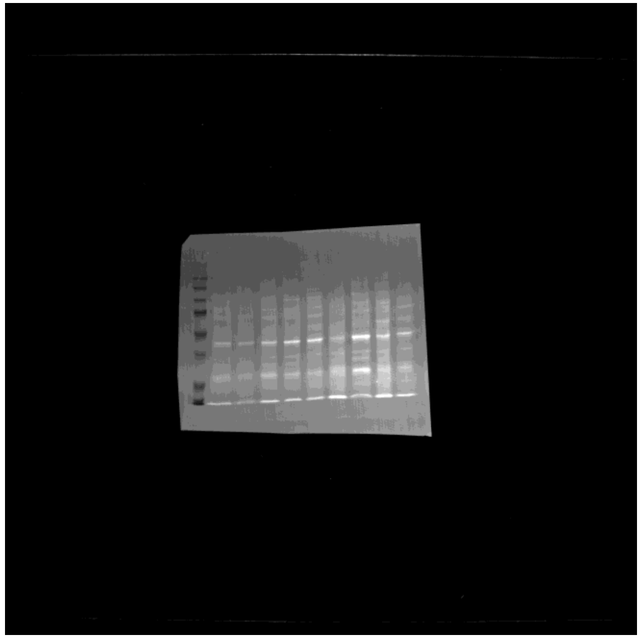

Supplement: Supplementary file 1 [file dermatopathology-12-00018-s001.zip › dermatopathology-3603760-supplementary.pdf]
